# Single-Trial Recognition of Video Gamer’s Expertise from Brain Haemodynamic and Facial Emotion Responses

**DOI:** 10.3390/brainsci11010106

**Published:** 2021-01-14

**Authors:** Ana R. Andreu-Perez, Mehrin Kiani, Javier Andreu-Perez, Pratusha Reddy, Jaime Andreu-Abela, Maria Pinto, Kurtulus Izzetoglu

**Affiliations:** 1 Faculty of Communication and Documentation, University of Granada, 18071 Granada, Spain; mpinto@ugr.es; 2 Centre for Computational Intelligence, School of Computer Science and Electronic Engineering, University of Essex, Colchester CO4 3SQ, UK; mehrin.kiani@essex.ac.uk (M.K.); javier.andreu@essex.ac.uk (J.A.-P.); 3 Sinbad2, University of Jaén, 23071 Jaén, Spain; 4 School of Biomedical Engineering, Science and Health Systems, Drexel University, Philadelphia, PA 19104, USA; ylr26@drexel.edu (P.R.); ki25@drexel.edu (K.I.); 5 Department of Sociology, University of Granada, 18071 Granada, Spain; jandreu@ugr.es

**Keywords:** mind-controlled games, brain signals, fNIRS, facial expressions

## Abstract

With an increase in consumer demand of video gaming entertainment, the game industry is exploring novel ways of game interaction such as providing direct interfaces between the game and the gamers’ cognitive or affective responses. In this work, gamer’s brain activity has been imaged using functional near infrared spectroscopy (fNIRS) whilst they watch video of a video game (League of Legends) they play. A video of the face of the participants is also recorded for each of a total of 15 trials where a trial is defined as watching a gameplay video. From the data collected, i.e., gamer’s fNIRS data in combination with emotional state estimation from gamer’s facial expressions, the expertise level of the gamers has been decoded per trial in a multi-modal framework comprising of unsupervised deep feature learning and classification by state-of-the-art models. The best tri-class classification accuracy is obtained using a cascade of random convolutional kernel transform (ROCKET) feature extraction method and deep classifier at 91.44%. This is the first work that aims at decoding expertise level of gamers using non-restrictive and portable technologies for brain imaging, and emotional state recognition derived from gamers’ facial expressions. This work has profound implications for novel designs of future human interactions with video games and brain-controlled games.

## 1. Introduction

Electronic sports (or eSports) is fast gaining acceptance as both at par with traditional sports, and the virtual athletes being celebrated as real-life sport athletes [1]. eSports is formally defined as ‘an area of sport activities in which people develop and train mental or physical abilities in the use of information and communication technologies’ [2]. The game League of Legends (LoL) is one of the most popular Multiplayer Online Battle Arena (MOBA) video games. The theme of the game LoL is fantasy combat strategy which, in 2019, had garnered an active player base of 80 million registered players, with 27 million active players on a per day basis [3]. The usage of eSports has been widely adopted as the new form of sports owing much to greater access to the internet [4].

Although there is a consensus to investigate the effect of the ever increasing usage of eSports, it is a rather complex task to quantify the eSports effect since there are varied genres, and sub-genres of video games, and also because how it affects one individual may not necessarily be the same for another individual. It has been established in previous studies that brain responses of individuals to a determined visual stimulus can vary significantly depending on their exposure to violent games [5]. Furthermore, the continuous use of such games is also found to have an impact on the behaviour, and personality of their users [6]. Also, significant correlations between the cognitive profiles and the neural substrates found in the neuroimaging analysis have been indicated in several neuroscience studies [7]. In this regards, a highly relevant aspect to investigate is the relationship between the proficiency (or the expertise) of participants in playing a game and their brain responses as well as emotional states.

**Figure 1:**
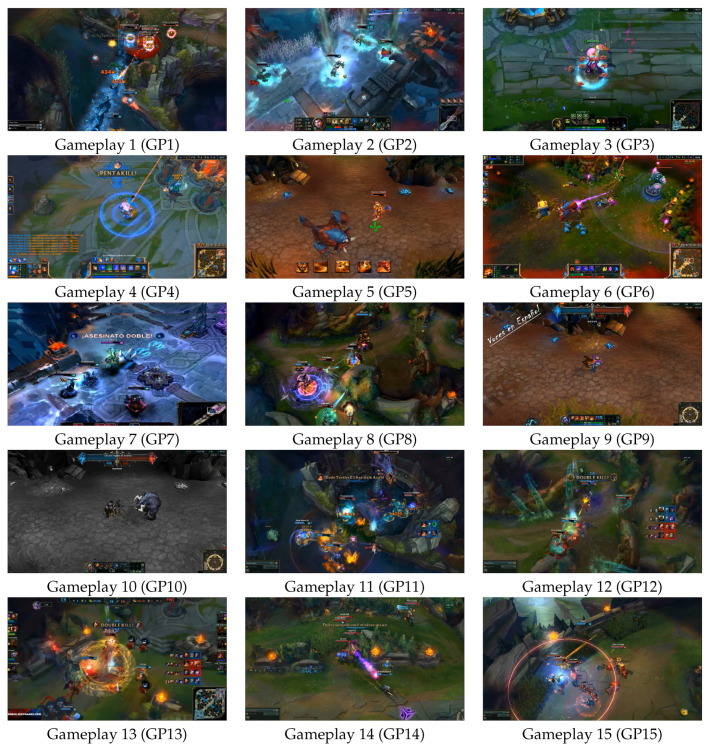
A representative image from all 15 gameplay (GP) videos used in the experiment.

**Figure 2:**
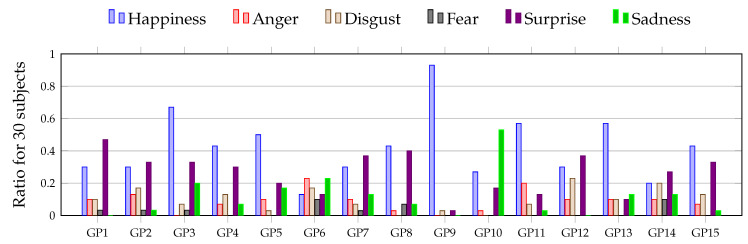
A bar chart representing the frequency of an emotion felt by participants as they watched the 15 gameplay (GP) videos used in this experiment. A representative image from all 15 GP videos are displayed in Figure 1.

Indeed, games are able to elicit a myriad of different cognitive processes and affective responses in players [8,9]. The same game, played for the same amount of time, can evoke varied physiological, psychological, and cognition-related responses across individuals [10]. In this work, the same phenomenon is also observed: 30 participants watched the same 15 gameplay videos from LoL, representative images from these 15 gameplay videos are shown in Figure 1, and a histogram from the self-reported variety of responses evoked in the 30 participants is shown in Figure 2. As can be readily appreciated from Figure 2, the same gameplay video is not evoking the same response across the 30 participants. However, despite gameplay evoking individual-specific responses, to the best of the author’s information, no direct attempt has been made in the literature to decode participant’s expertise using brain as well as emotion responses to gameplay captured by nonrestrictive bio-signal monitoring.

Converging from all these evidences, the present novel study aims to automatically recognise gaming expertise of participants by capturing elicited neural responses from prefrontal cortex (PFC) using functional Near-Infra red Spectroscopy (fNIRS). In addition, the effect of integrating a measure of affective state, like emotion decoded from participant’s facial expressions, in such a recognition system would also be investigated. In the present study, we have bench-marked several classification methods and managed a satisfactory recognition of expertise in several episodes of the game LoL. The episodes of the game LoL were identified by other players, external to the participants’ sample, as pertaining to different emotional context (for instance, exciting, extraordinary, funny, violent, stressful and sad episodes of the game LoL).

In particular, the present study aims at answering the following research question: to what extent is it possible to recognise gamer’s expertise from their brain responses to gameplay videos, what methods provide a better accuracy and to evaluate the effect that emotions recognition through participant’s facial expression analysis can have in bolstering expertise recognition. This research question is of special interest for the game industry [11].

To this end, we test the following three valid hypotheses $H_{1}$ and corresponding null hypotheses $H_{0}$:

- **$H0_{1}$**: It is not possible to recognise gamer’s expertise level from fNIRS neural responses from the PFC with recognition performance (measured as cross-validated f-scores) better than a 95% confidence interval difference to the results from a baseline (rule 0) classifier.
- **$H1_{1}$**: It is possible to recognise gamer’s expertise level from fNIRS neural responses from the PFC with recognition performance better than a 95% confidence interval difference to the results from a baseline (rule0) classifier.
- **$H0_{2}$**: Assert $H1_{1}$ and the adding of facial expression emotions **does not bolster** the recognition of expertise with statistical difference of p<0.05.
- **$H1_{2}$**: Assert $H1_{1}$ and the adding of facial expression emotions **bolsters** the recognition of expertise with statistical difference of p<0.05.
- **$H0_{3}$**: Assert $H1_{1}$ and/or $H1_{2}$ and **there are not** statistical differences of performance (α=0.05) in the recognition form each benchmarked classifier.
- **$H1_{3}$**: Assert $H1_{1}$ and/or $H1_{2}$ and **there are** statistical differences of performance (α=0.05) in the recognition form each benchmarked classifier.

The main contributions and findings of this novel study are:

1. This work successfully recognizes the expertise level of gamers with high accuracy (91.44%) using brain signals acquired from a portable neuroimaging modality (fNIRS).
2. This is also a first study that integrates brain signals with the emotional states of the participants, derived from the visual cues provided by their facial expressions, in the classification paradigm to investigate the interlink between the emotional state of a participant and their expertise level.
3. This work also found that unsupervised deep feature extraction for time-series boosted recognition performance specially when applied to fNIRS signals.

The findings of this work pave the way for new avenues of interacting with games, such as affective brain-to-game interfacing for entertainment or serious games. This novel way of profiling of gamers can enable mind-to-game interlinked experience allowing a higher coupling between the real expertise of the user, and the level reached in the game.

The paper is organised as follows: In Section 2: Background, an overview on affective gaming and the successful usage of fNIRS modality in various gaming studies is presented. This is followed by Section 3: Related Works where different physiological markers driving the online game development are discussed alongside similar studies that have decoded the manual dexterity of participants using their brain activity imaged with fNIRS in different real-life settings. Section 3 also entails a review of studies investigating the effect of gameplay videos on gamers’ cognition. In Section 4 Materials and Methods, the outline of the experiment, along with data collection and subsequent feature engineering stages are presented. Section 4 also covers the classification paradigms in detail. Results are presented in Section 5 followed by a conclusion in Section 6.

## 2. Background

With the increased consumption of eSports and the availability of sophisticated technology, there is an increasing trend of modifying game themes in real time to enhance a user’s experience. In this section, we discuss how varying feedback from reading users physical and mental state, for example fNIRS for reading brain activity and emotional state of the gamers perceived from their facial expressions, is being used to modify the game content to enhance their overall gaming experience.

### 2.1. Mind-Controlled Games

Traditional video gaming involve playing games in a simulated environment with users interacting with peripheral devices such as mouse, keyboard, and/or joysticks. However, with the availability of more sophisticated technology, now users are also able to interact in real-time with video games using their facial expressions, body movements, and even physiological signals like heart rate, skin conductivity etc. [12]. An interesting area is to directly monitor cognitive processes through Brain Computer Interfaces (BCI) to control game function [13,14]. Beyond controlling the game physics as in BCI, integrating gamers’ cognitive experience as a higher induction of cognition permits new kind of physiological computing [15,16] in video games.

### 2.2. On Game Experience and Sensors

In recent years, researchers have been focusing on finding the link to gamer’s experience in lieu with their physiological markers to drive the online game development content towards enhancing the overall gamers experience. The work by Drachen et al. [17] focus on learning about gamer’s experience using correlation between user experience and physiological measures (electrodermal activity and heart rate). They conclude that a high level of engagement, both positive e.g., excitement, and negative e.g., frustration, can be established based on high correlation with physiological measures. There seems to be a direct indication of how a user felt while playing a game based on their physiological measures.

Another important consideration when creating an emotionally adaptive game is behavioural expressions. In the work by [18], the link between behavioural expressions such as patterns in pressure on mouse, postural movement and game involvement is established. The game difficulty level is varied, and the values of behavioural expressions are recorded. They concluded that as the level of game difficulty was increased, the level of frustration or enjoyment, taken together as engagement, also increases.

### 2.3. On fNIRS during Video Gaming

The role of functional Magnetic Resonance Imaging (fMRI) in establishing the functions of different brain regions before, and after learning, remains pivotal. However, for neuroergonomic studies in specific, which focus on understanding the human brain functions in everyday life settings, the confinement of a scanner environment is not ideal. In this regard, fNIRS, another optical neuroimaging modality, is fast emerging as a de-facto choice for imaging the human brain for neuroergonomic studies owing to its non-invasive, highly portable and wearable characteristics [19,20]. fNIRS uses near infra-red (NIR) light to read cerebral activity by introducing NIR at the specific location of interest over the scalp. The brain activity is measured by fNIRS in terms of the changes of cortical deoxygenated, and oxygenated haemoglobin (oxyHb) concentrations [21].

In the study by Cakir et al. [22] fNIRS was successfully employed to investigate the changes in brain activation in PFC owing to playing a genre serious mobile game. Similarly, in the study by Bunce et al. [23] the relation between a participant’s level of expertise and their task performance using fNIRS is investigated. They conclude that brain activations differ for participants with varying levels of expertise.

Another study which focused on gauging whether the effect of a decrease in oxyHb in dorsal PFC on playing video games for a long time is also exhibited by young children aged 7–14 years is reported here [24]. They conclude that a decrease in attention of the user when they have been playing a video game for a long duration of time is a phenomenon exhibited by both adults, and children, alike.

## 3. Related Works

In this section we outline relevant works that investigated how playing video games impact gamers’ cognition, and further how measurements from brain, in particular using fNIRS, have been previously made use of for decoding manual dexterity of users. All of the research evidence provided in the following subsections helped us frame the research question of the present study.

### 3.1. Game Experience and Cognition

To gauge the effect on playing video games on cognition and behaviour, a notable review is presented in [10]. They divulge on the various facets of the effect of playing video games on an individuals’ abilities. Although they all agree that given the diversity of the video game genre’s and settings, it is not possible to give a conclusive yes/no answer to its effects, but there are some established advantages and disadvantages that have come forward.

Some of the possible effects of playing video games can be attributed to practise in the parallel of real-world games. The practise of paying attention, staying focused, for longer duration of times has proven to improve visual skills in individuals with amblyopia (lazy eye) [10].

In the study by Hyun et al. [25], they establish that participants who take part in a regular, long-term playing of games have a consequent incremental change in anatomical volume in their PFC. Another related study by Gong et al. [26], reported a similar result that for those gamers who played for less time have decreased brain activity indicating that playing video games for longer times results in overall increase in cognition of participants.

Another notable study trying to draw the link between intelligence and expertise in playing video game is done by Kokkinakis et al. [27]. They argue that as we associate a higher ability level of those who are experts in playing real world games (like Chess, or Mancala), likewise experts in video games, which are built as games of strategy, should also be regarded as those who possess a higher level of intelligence quotient. They base their analysis on an online game MOBAs. A higher performance in MOBA is based on a combination of skills like memory, tactics, attention, and strategy—the different facets of fluid intelligence.

Although in these notable works some behavioral and/or anatomical differences were found in participants on account of playing video games, these studies did not encompass a direct recognition of participants expertise from their functional bio-signals, as intended in this present study.

### 3.2. Expertise with fNIRS

The neuroimaging modalities most commonly utilised for measuring brain activity in BCI applications are fNIRS [28] and electroencephalogram (EEG) [16,29] since these modalities are non-invasive, portable, and allow greater flexibility for participants posture (e.g., sitting upright) 180 whilst recording their brain activity. In this work, fNIRS is used to image participant’s brain activity owing to the superior spatial resolution and resistance to motion artifacts in comparison to EEG [30]. In addition, previous fNIRS studies from our group have aimed to gauge technical skill levels assessment for surgeons with varying levels of experience in the study by Andreu-Perez et al. [28] and Kiani et al. [31]. In Andreu-Perez et al. [28] the surgeons perform a complex bi manual coordination task whilst their brain activity is recorded using fNIRS. Their results demonstrate that it is possible to classify operator skill level from functional connectivity data with statistical significance. However, they garnered brain activity from only three customary surgical needling tasks resulting in a classification accuracy of 82% for time-course based networks, and 68% for session based networks. However, both studies [28,31] did not take into account any aspect of the emotional state of their participants whilst decoding levels of manual dexterity.

Another study that investigated the relation between changes in mental workload, level of expertise, and task performance for aerospace application on brain activity recorded using fNIRS is by Ayaz et al. [32]. They concluded that fNIRS signal measurements are correlated to task performance, and subjective self-reported measures. In addition, they did not extend their analysis to decode the expertise level of the participants based on their brain activity.

Indeed, the success of decoding participant’s skill level directly from brain activity recorded using fNIRS, used in tandem with emotional state of the participants, has the potential to conduct objective assessment of particpants whilst they perform a task in more naturalistic settings. The feedback from such a decoding system may also be used to improve operator performance during technical skill training. Hence towards this end, in this work, we aim to decode gamers expertise level using their brain activity recorded with fNIRS as well as emotional state derived from facial expressions.

## 4. Materials and Methods

### 4.1. Experimental Details

In this work, a total of 30 participants, casual and professional gamer’s, of varying expertise levels were recruited to visualise 15 gameplay videos taken from the game LoL. This gives a total of 450 trials (30 participants * 15 trials = 450 trials). This study was conducted following Declaration of Helsinki norms and approval by a local Ethics Committee on Human Research (CEIH) and The International School for Postgraduate Studies (Ref.: UGR24102017). Written consent was obtained from all participants. A representative image from all 15 gameplay videos which the participants watch in the experiment are shown in Figure 1. Whilst the participants are performing the experiment (i.e., watching one of the 15 gameplay videos from the game LoL) their brain activity is recorded using a fNIRS sensor, and simultaneously the face of the participants is also video recorded to capture their emotional state vis-a-vis facial expressions.

**Figure 3:**
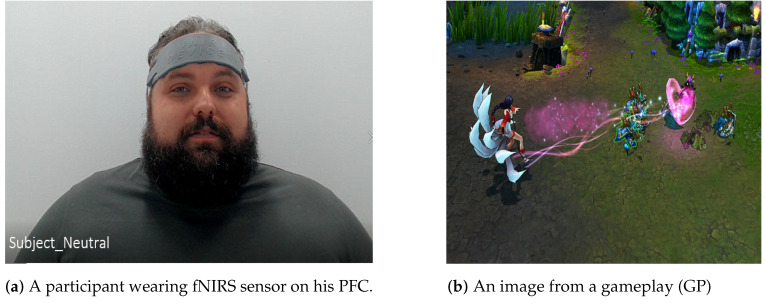
(**a**) A participant watching a (**b**) gameplay (GP) video whilst his brain activity is recorded using a fNIRS sensor placed on prefrontal cortex (PFC).

The motivation of the experiment is to be able to ascertain the expertise level of the participants in playing the game LoL using only their brain data, and emotions predicted scores derived from their facial expressions. In order to achieve this goal (i.e., determine a participant’s expertise level using their brain data, and emotion scores) a fNIRS sensor is placed on PFC of each participant as can also be seen in Figure 3. The reason for recording brain activity of all participants from PFC is because it is the part of brain associated with concentration and planning [33]. Therefore, fNIRS sensor placed on PFC will record the differences in the concentration of haemoglobin molecules in PFC region for users with varying expertise levels. The variation in the fNIRS data, owing to different expertise level of participants, will become the basis for classification of their expertise level.

In order to also establish the link between the expertise level and the emotional state of participants while they are performing a given experiment, a video of their facial expressions is also recorded. To the best of the author’s information, there is no established correlation between the expertise level and the emotions of a gamer in the literature. This is why, in this work, a preliminary investigation is performed to see if the additional information from estimated emotions scores from the facial expressions would give further insights that would bolster the classification for expertise of gamers.

The 30 participants are grouped in three main expertise groups: as 10 *novices* (NVs), 9 *intermediates* (ITs), and 11 *experts* (EXs). The criterion used to classify a participant in one of the three given expertise categories (NV or IT or EX) is based on the average victory points accumulated by the participant, and the average number of hours per week the participant plays the game LoL, as outlined in Table 1. A Mann-Whitney test [34] is performed on the victory points for the classification of participants, and the distributions for NVs, ITs, and EXs, are ascertained to be statistically different. For more details on the group level criterion, please see the earlier work here [35].

### 4.2. fNIRS Data

The fNIRS data is recorded using a commercially available research-grade fNIRS system (fNIR Devices LLC, Potomac, MD, USA). The 16 fNIRS channels on the fNIRS sensor have 8 channels on the right PFC, and 8 on the left PFC.

The fNIRS data collected from the fNIRS sensor is pre-processed in 3 stages:

1. The data from any channel that fails to meet the set pass criterion is eliminated from subsequent data analysis. The most common reasons for failing to meet the pass criterion were significant light leakage, low signal levels, and/or saturated signals.
2. The data from the passed channels is low pass filtered (cut off frequency 0.14 Hz) and compared with baseline signal to compute change in optical density using modified Beer-Lambert law.
3. Outlier data beyond the 3 standard deviations from the mean is removed before any features are calculated.

Overall 12% of the fNIRS data failed to meet the quality checks listed above and was excluded from the study. For more details on the fNIRS data pre-processing stages, please see the earlier work here [35].

### 4.3. From Facial Expression’s Data to Emotion Scores

A video of the face of the participants performing the experiments is also recorded in tandem whilst their brain activity is recorded using fNIRS. The reason the participants are recorded while performing the experiments is to record their facial expressions in order to subsequently use them in combination with the brain data to determine their expertise level group.

**Figure 4:**
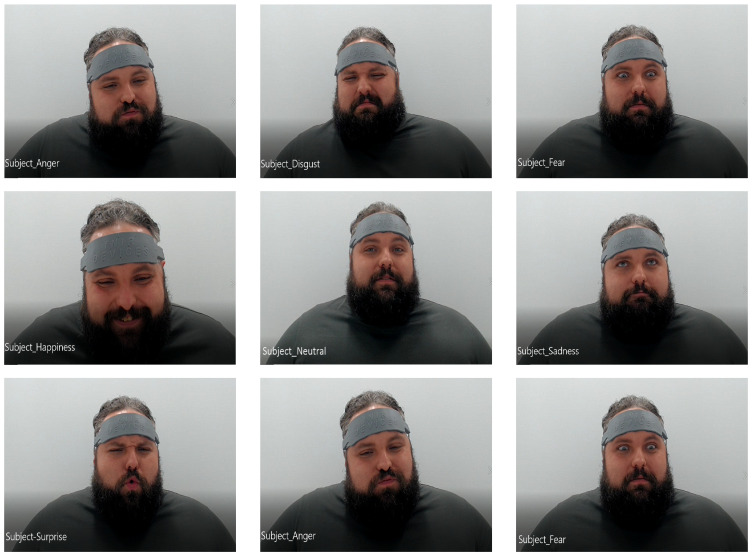
An array of representative facial expressions of a participant’s seven emotions: Anger, Disgust, Fear, Happiness, Sadness, Surprise, and Neutral. The participant gave his consent to share his images.

All videos of the participants for each trial are split into 18 video frames. Each frame of the participant’s video is then categorized into one of the following seven emotions: Anger, Disgust, Fear, Happiness, Sadness, Surprise, Neutral. A pictorial compilation of all of the aforementioned facial expressions from emotions for one subject is shown in Figure 4. The self-reported varied emotions aroused in the participants when they watched the 15 gameplay videos from LoL (also depicted in Figure 1) are shown as a bar chart in Figure 2.

The participants’ video for each of the 15 trials, one trial for each of the 15 gameplay videos from LoL, is sampled twice per second. Each gameplay had a duration of about 30.26 ± 14.33 s. The total number of frames included from the video of each trial is 18 so that all trials have the same consistent number of frames. The facial emotions classifier used for categorising each video frame is VGG19 [36]. The classification model VGG19 is a convolutional neural network based PyTorch implementation on facial expression recognition. It is trained for the dataset Facial Expression Recognition 2013 (FER-2013) [37]. The classification accuracy of VGG19 on FER-2013 is 73.112% (10-fold cross-validation).

The participant’s expressions from the gamers’ experiment in this work are not used to train the emotions recognition model. The intent is to use an off-shelf model that is already trained using thousands of images of human facial expressions as a universally trained model to be used on any human facial expression dataset. Each of the 18 video frames for a given trial is then given emotion prediction score by FER-2013. For example, the prediction score [0.0424,0.0024,0.0177,0.8275,0.1035,0.0013,0.0051] for a video frame corresponds to facial emotional expressions of Angry, Disgust, Fear, Happiness, Sadness, Surprise, Neutral respectively. The highest prediction score is for the expression of emotion Happiness for the given video frame.

### 4.4. Classification Paradigms

In this section, supervised and unsupervised classification paradigms are explored to establish which combination of techniques allows for maximum classification accuracy for determining the expertise level of the gamers. The classification paradigms are investigated with two datasets (DSs):

1. DS1: Brain time-series data acquired from fNIRS alone.
2. DS2: Brain time-series data acquired from fNIRS appended with predicted emotion scores, on participant’s expression data obtained from VGG19, on a per trial basis.

**Figure 5:**
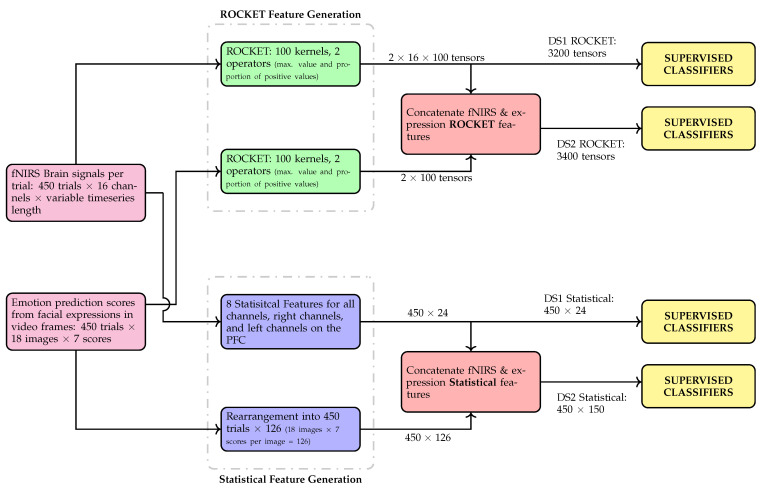
Flowchart of the different multi-modal data frameworks used in this study for comparison purposes, feature generation with hand-crafted features (statistical) or ROCKET and classification of expertise level per trial.

The motivation for using the two aforementioned DSs is to establish whether accuracy of expertise classification is improved when fNIRS data is used in tandem with predicted emotion scores data (DS2) in comparison to when only fNIRS data is used (DS1). In addition, the classification prowess of the supervised classifiers are also gauged when these classifiers are given statistical features in comparison to random convolutional kernel transform (ROCKET) [38] features. A flowchart depicting the overall classification paradigm is presented in Figure 5. More details on the feature generation, both statistical and ROCKET features, and the classifiers used are presented in the following sections.

#### 4.4.1. Statistical Feature Learning for Time Series

A total of 8 statistical features are computed for individual fNIRS channels (1–16), ‘All’ channels (Average of all passed channels), ‘Right’ channels (Average of passed channels located on the right PFC), ‘Left’ channels (Average of passed channels located on the left PFC). The 8 features computed per trial are: Mean, Standard Deviation, Maximum, Minimum, Range, Signal Slope, Skewness, and Zero-crossing. These features are computed for each of the total 450 trials. This results in dataset (DS1) of size 450 (trials) by 24 (statistical features) as also illustrated in Figure 5.

#### 4.4.2. Unsupervised Deep Feature Learning for Time Series

In this work, both DSs time series feature learning is done using ROCKET [38]. A set of convolutional kernels are defined by their length, weights, bias, dilation, and padding. However, in ROCKET implementation the only hyper parameter that needs to be defined manually is the number of kernels, and it works in an unsupervised fashion. All other ROCKET hyper parameters (i.e., length, weights, bias, dilation, and padding) are sampled randomly i.e., each kernel of ROCKET assigns a random number for its length, weights, bias, dilation, and padding. In this work we used 100 kernels, with 2 operators: (1) maximum value operator and (2) proportion of positive values operator, as defined in [38]. Resultant feature dimensions from this processing are depicted in Figure 5.

#### 4.4.3. Supervised Classifiers

A total of 7 supervised classifiers of varying genres are used with both DS1 and DS2 each separately with both statistical and ROCKET features as shown in Figure 5. The classifiers used, along with the detail of their hyper parameters is given below. The same hyper parameters are used for both DSs so that any difference in the classification accuracy for the two DS by a given classifier can be attributed to the inclusion of emotion scores in DS2.

- *Random Forest (RF)*: A RF is a ensemble learning method that incorporates multiple decision tree classifiers on varying sub-samples of the input data set. RF improve the predictive accuracy, and control over-fitting by averaging the decision of the multiple decision tree classifiers [39]. In this work the number of multiple decision tree classifiers used are 30, each with a maximum depth of 10. We used the implementation in *Scikit-learn* [39] library.
- *Support Vector Machines (SVM):* SVM is inherently a supervised discriminative classifier constructed by a separating hyper plane in a multi-dimensional space [40]. SVM optimize the hyper plane definition by maximizing the distance to the nearest data point of any class. In this work, non-linear kernel *radial basis function* is used with gamma set at *auto*. We used the implementation in *Scikit-learn* [41] library.
- *k-Nearest Neighbors (kNN):* kNN capitalizes on the similarity idea that data points from a given class would have more similarity with each other in comparison to data points from different classes. The measure of similarity can be computed using different metrics such as distance, proximity, or closeness [42]. The number of neighbors used in this work for computing the similarity is 3. We used the implementation in *Scikit-learn* [41] library.
- *Gaussian Naive Bayes (GNB):* GNB is an effective inductive learning algorithms based on applying Bayes’ theorem. The term ‘naive’ appears because of the underlying assumption of conditional independence between every pair of features used in the implementation of GNB [43]. In this work, the default parameters are used for GNB. We used the implementation in *Scikit-learn* [41] library.
- *XGradient Boost (XGB):* XGB provides parallel tree boosting machine learning algorithms using an optimized distributed gradient boosting library designed to be highly efficient, flexible and portable [44]. In this work, the XGB objective is set to *multiclass* with fraction of columns to be subsampled as 0.3, learning rate as 0.1, maximum depth as 3, alpha as 5, nestimators as 15, and numclass as 3. We used the implementation in *Scikit-learn* [41] library.
- *Fully Connected Deep Neural Network (FCDNN):* The FCDNN consists of 5 blocks. Each block consists of:
  - Fully connected (FC) layer.
  - Rectified linear unit (ReLU) activation.
  - Each FC layer also dropouts neurons to ensure FCDNN is not over fitting with any of the DSs.

Table 2 enlists the optimized hyper parameters of FCDNN for both DS1 and DS2. The number of epochs the FCDNN is trained on is 1000. The optimizer used is ADAM and the learning rate values for DS1 is $3.95\cdot 10^{-5}$ and for DS2 it is $8.28\cdot 10^{-5}$. This was implemented with custom code in *PyTorch* 1.3.1 [45] library.

**Figure 6:**
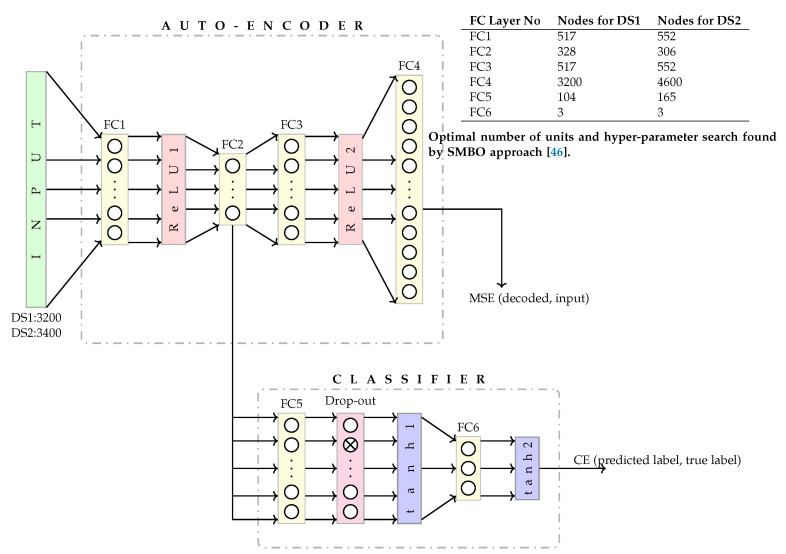
Deep Classifier AutoEncoder (DCAE) architecture with optimal number of units and hyper-parameter search found by SMBO approach [46].

- *Deep Classifier Auto Encoder (DCAE):* The architecture of the DCAE along with its hyper parameters i.e., the number of nodes for each layer, dropout layer rate, for both DS1, and DS2 are shown in Figure 6. It consists of an encoder, decoder, and a classifier. This was implemented with custom code in *PyTorch* 1.3.1 [45] library.

The number of epochs the DCAE is trained on is 1000. The optimizer used is ADAM and the learning rate values for DS1 is $4.14\cdot 10^{-5}$ and for DS2 it is $4.87\cdot 10^{-5}$.

The structure of the auto-encoder which consists of an encoder and a decoder is delineated as follows:

- Encoder: The number of inputs to the encoder are 3200 inputs for DS1 and 4600 inputs for DS2. The encoder consists of a fully connected layer (FC1), Rectifier Linear Unit (ReLU1) activation, and FC2.
  - Fully Connected Layer (FC1): A fully connected layer that performs a linear transformation (i.e., a weight matrix and bias) with ReLU1 activation. It has 517 hidden units that encode 3200 inputs for DS1, and 551 hidden units to encode 4600 inputs for DS2.
  - Fully Connected Layer (FC2): A fully connected layer that performs a linear transformation with 328 hidden units for DS1, and 306 units for DS2. The output of FC2 is the output of the encoder.
- Decoder: The number of inputs to the decoder are 328 inputs for DS1 and 306 inputs for DS2. The decoder consists of FC3, ReLU2 activation, and FC4.
  - Fully Connected Layer (FC3): A fully connected layer that performs a linear transformation with 517 hidden units for DS1, and 551 hidden units for DS2, with ReLU2 activation that decode from 328 hidden units for DS1, and 306 units for DS2.
  - Fully Connected Layer (FC4): A fully connected layer that performs a linear transformation which decode 517 encoded inputs to 3200 decoded inputs for DS1, and decode 551 encoded inputs to 4600 decoded inputs for DS2. The output of FC4 is the output of the decoder.

The error between the decoded inputs, and the inputs from DS1 (or DS2) is computed using mean square error (MSE).

- Classifier: The input to the classifier is the output of FC2 i.e., 328 inputs for DS1 and 306 inputs for DS2. It consists of FC5, drop-out, hyperbolic tangent activation function (tanh1), FC6 and tanh2 layers, as outlined below:
  - Fully Connected Layer (FC5): A fully connected layer that performs a linear transformation with 328 inputs, and 104 outputs for DS1, and 306 inputs and 165 outputs for DS2.
  - Dropout: FC5 output undergoes 39.22% dropout for DS1, and 49.96% for DS2 followed by hyperbolic tangent activation function (tanh1).
  - Fully Connected Layer (FC6): A fully connected layer that performs a linear transformation with 104 inputs for DS1, and 165 inputs for DS2, and 3 outputs followed by hyperbolic tangent activation function (tanh2).

The error between the predicted label of the expertise level of a participant (from FC6), and the true expertise level is computed using cross entropy (CE) loss.

The hyper-parameters of FCDNN, and DCAE, which include number of hidden units for each FC layer, dropout rate, and learning rate, are optimized using a sequential model-based optimisation based on a tree-structured Parzen estimator search algorithm [46]. In order to keep a check on bad trials, a custom termination condition is also implemented that terminates a trial if the loss reduction is less than $10^{-5}$ for every 100 epochs.

## 5. Expertise Classification Results

The expertise classification results are obtained both separately for each trial for DS1 and DS2. For each of the 7 classifiers, the classification results are reported for 10 repeated stratified k-fold cross-validation (CV) with 5 splits DS1, and DS2. The data was split with 20% as test and the remaining as training and validation. The mean and standard deviation of the statistical metrics of accuracy, precision, recall, and F1-scores for the classifiers are reported in Table 3a for DS1, and Table 3b for DS2. For both DS1 and DS2, the best classification accuracy has been obtained by the classifier using DCAE at 90.70 ± 7.84% for DS1, and for DS2 the best classification accuracy is obtained by FCDNN at 91.44 ± 6.32% for DS2.

Also, for both DSs, the classification performance of deep neural networks i.e., FCDNN and DCAE is considerably better than other machine learning algorithms i.e., RF, SVM etc. One possible explanation for this can be that the deep learning architecture further enables the discovery of relevant patterns automatically from the enhanced features provided by ROCKET for both DSs. Although there is minimal difference in accuracy metrics for FCDNN and DCAE, the convergence and optimisation happens earlier for DCAE because its range of search and number of hyper-parameters is smaller. Also, the inclusion of the autoencoder helps to reduce the unnecessary dimensions for the classifier. It is worth mentioning that the parameters for the autoencoder and classifier in DCAE are jointly learned as both loss functions are added and back-propagated through the layers.

**Figure 7:**
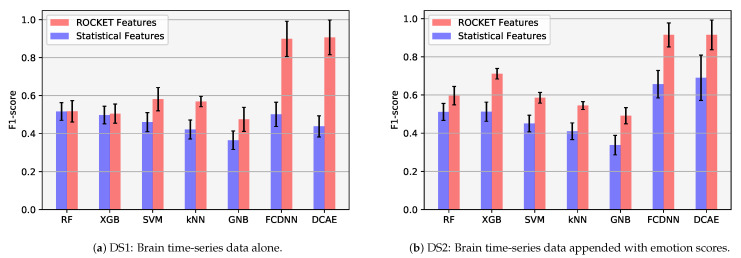
Comparison of mean F1 scores with standard deviation for all classifiers per trial for (**a**) DS1 and (**b**) DS2 using ROCKET and statistical features.

A comparison of which feature set to use, statistical features or ROCKET features, is best suited for the expertise recognition from bio-signal data is also drawn on Figure 7. In Figure 7, a bar chart for the F1 scores for all classifiers for DS1 and DS2 using both statistical features and ROCKET features indicate a clear trend that the classification prowess of the classifiers is improved significantly when the input feature set is ROCKET (in comparison to statistical features).

Across all classifiers, the maximum increase in classification accuracy on inclusion of expression data (i.e., the difference between DS1 and DS2) is for XGB with a percentage increase in classification accuracy by 40.87%. To appreciate the improvement in classification accuracy on inclusion of emotion scores, a bar chart for the F1 scores for all classifiers for both DS1 and DS2 is also plotted in Figure 7. As can be seen in Figure 7, the height of the bars on the right hand sides bar graph is greater, in comparison to those on the left hand side, for most of the classifiers, indicating an overall trend of increase in the classification prowess of the classifiers on inclusion of emotion scores. Nevertheless, this improvement is less pronounced for deep models, which sustain a satisfactory level of recognition with brain data only as well. Overall, the increase in F1-scores is only slight for most classifiers, and as is also reported in Table 4b not statistically significant for all classifiers.

### 5.1. Hypotheses Testing

In this section the test for the three hypotheses’ outlined in Section 1 are presented here. For hypothesis test we use non-parametric Kruskal-Wallis (KW) [47] test, and correction Dunn-Sidak [48] for multi-comparisons is applied, at significance level α=0.05.

#### 5.1.1. $H0_{1}$

To test the $H0_{1}$, a Kruskal-Wallis (KW) test on F1-scores obtained for 10 repeated stratified k-fold CV with 5 splits from all classifiers are compared with a baseline classifier (baseline classifier from the Scikit-learn [41] library) at significance level 0.05. The F1-scores for baseline classifiers are also obtained for 10 repeated stratified k-fold CV with 5 splits. By comparing the F1-scores for the classifiers used in this work with those of baseline classifer, we can test the $H0_{1}$ that it is not possible to recognise gamer’s expertise level from fNIRS neural responses from the PFC with recognition performance better than a 95% confidence interval difference to the results from a baseline classifier. The results of the KW test are presented in Table 4a.

For all classifiers, except RF, $H0_{1}$ is rejected. Hence, for six out of a total of seven classifiers the $H1_{1}$ is accepted that it is possible to recognise gamer’s expertise level from fNIRS neural responses from the PFC with recognition performance better than a 95% confidence interval difference to the results from a baseline classifier.

#### 5.1.2. $H0_{2}$

In order to test $H0_{2}$ the F1-scores distributions of each classifier for DS1 and DS2, a KW test is performed at significance level 0.05. For each classifier, the F1-scores are obtained for 10 repeated stratified k-fold CV with 5 splits DS1, and DS2. The results of the KW test for $H0_{2}$ for both DS1 and DS2 for each classifier are reported in Table 4. For four classifiers (RF, XGB, SVM, kNN), the $H0_{2}$ can be rejected hence for these four classifiers the $H1_{2}$ is accepted that adding facial expression emotions decoding bolsters the recognition of expertise.

#### 5.1.3. $H0_{3}$

In order to test $H0_{3}$ KW is performed at significance level 0.05. The KW test results with p-values, lower and upper bound are reported in Table 5a for DS1, and Table 5b for DS2. For all classifiers, $H0_{3}$ is rejected for at least four out of a total of seven classifiers.

## 6. Discussion and Conclusions

With the ever-increasing consumption of eSports, it is pertinent to explore how it is affecting the gamers, and watchers alike. In particular, in this study, we explored two main aspects in which greater consumption of eSports can affect the gamers- mainly (i) the neuroplasticity of the brain in response to 460 gaining expertise in playing a game, and (ii) the emotional state of a gamer. Previous studies have demonstrated that the expertise level of participants can lead to a change in brain activity in response to experience, also known as neuroplasticity [32], and continuous consumption of such eSports may effect on the cognition and emotional states of gamers [49]. Hence, to gain an insight into the link between an emotional state of a gamer, and their expertise, classification analysis with only fNIRS data (DS1) and classification analysis with fNIRS data in tandem with derived emotions from facial expressions (DS2) is performed separately. Also, a range of supervised classifiers have been explored with feature extraction strategies for both DS1 and DS2.

The best classification accuracy is obtained by DCAE classifier at 90.70 ± 7.84% for DS1 with ROCKET features. For DS2 the best classification accuracy is obtained by FCDNN at 91.44 ± 6.32% with ROCKET features. For six out of a total of seven classifiers, the $H0_{1}$ is rejected hence establishing that it is possible to recognise gamer’s expertise level from fNIRS neural responses from the PFC with recognition performance better than a 95% confidence interval difference to the results from a baseline classifier. This is inline with previous studies which found professional on-line gamers to have an increased brain volume in the PFC [25], and from the literature we know that subjects at different 475 expertise levels have different patterns of activation in their PFC [28,31].

A comparison of the F1-scores for all the classifiers for DS1, and DS2, also plotted in Figure 7, indicate that for six out of a total of seven classifiers explored, the classification accuracy improves with DS2 i.e., when a classifier is also given the information from the emotions of the gamers. However, we did not find the increase in accuracy to be statistically different for all classifiers, as also reported in Table 4b. Nevertheless, this warrants further investigation perhaps with a greater number of participants (the current study has *n* = 30), and a selection of gameplay that evokes stronger emotional responses from the participants.

In addition our work has demonstrated that the performance of all classifiers, for both DS1 and DS2, improved markedly when recognising expertise level of gamers using ROCKET features in comparison to hand-crafted (viz. statistical) features. The classification results from all classifiers are also tested for $H0_{3}$ that there are not statistical differences of performance α=0.05 in the recognition form each bench marked classifier. As reported in Table 5, $H0_{3}$ is rejected for all classifiers for at least four other classifiers.

Overall, this work has demonstrated successful classification of gamer’s expertise level using their brain data, and emotions decoded from facial expressions. We expect that the findings of this work pave the way of new designs of affective and mind-controlled gaming. For example, integration of neural data in games, such as identification of gamers’ expertise based on their neural responses achieved after time played, rather than discrete counts of points achieved after game played.

## Figures and Tables

**Table 1:** Classification of participants into one of the three expertise levels based on their accumulated victory points, and the number of hours spent per week playing the game *League of Legends*.

Expertise Level	Victory Points	Hours Played Per Week
Novices (NVs)	0–400	{h|h≤1}
Intermediates (ITs)	200–400	{h|1<h≤3}
	400–1000	{h|3<h≤5}
Experts (EXs)	More than 1000	{h|h>5}

**Table 2:** Optimal hyper-parameter values of FCDNN for DS1 and DS2.

FC Layer No.	Hyper Parameters	DS1	DS2
FC1	Number of nodes	244	244
	Dropout	0.333	0.231
FC2	Number of nodes	313	148
	Dropout	0.344	0.433
FC3	Number of nodes	160	119
	Dropout	0.453	0.265
FC4	Number of nodes	149	102
	Dropout	0.346	0.391
FC5	Number of nodes	65	182
	Dropout for FC5	0.395	0.691

**Table 3:** Single-trial classification results for expertise levels of participant’s into NVs, ITs, and EXs (**a**) DS1 and (**b**) DS2 with ROCKET features or statistical features using various classification paradigms.

(**a**) Classification results for DS1: Brain data acquired from fNIRS alone.					
**Classifier**	**Features**	**Accuracy**	**Precision**	**Recall**	**F1-Score**
RF	ROCKET	52.06 ± 5.56	53.95 ± 10.92	51.77 ± 12.17	51.72 ± 5.63
	Statistical	51.69 ± 4.95	52.13 ± 7.74	51.39 ± 10.09	51.43 ± 4.96
XGB	ROCKET	50.79 ± 4.99	52.39 ± 8.87	50.47 ± 10.96	50.50 ± 5.10
	Statistical	50.51 ± 5.05	51.86 ± 9.20	50.05 ± 10.62	50.23 ± 5.10
SVM	ROCKET	58.23 ± 6.07	62.02 ± 14.06	57.72 ± 12.91	58.11 ± 6.20
	Statistical	46.60 ± 4.92	46.97 ± 8.12	45.66 ± 14.34	45.69 ± 5.04
kNN	ROCKET	56.89 ± 3.06	59.87 ± 12.11	56.62 ± 10.94	56.87 ± 2.78
	Statistical	42.44 ± 4.18	43.85 ± 8.12	42.11 ± 11.63	41.97 ± 4.34
GNB	ROCKET	47.86 ± 6.86	50.45 ± 10.62	47.27 ± 12.34	47.49 ± 6.38
	Statistical	41.49 ± 3.70	46.29 ± 13.90	39.27 ± 30.91	35.95 ± 4.29
FCDNN	ROCKET	89.84 ± 9.31	89.93 ± 9.19	89.84 ± 9.31	89.84 ± 9.28
	Statistical	51.20 ± 6.07	50.40 ± 6.14	52.89 ± 7.02	50.10 ± 6.38
DCAE	ROCKET	90.70 ± 7.84	90.65 ± 8.65	90.70 ± 7.62	90.64 ± 9.14
	Statistical	45.97 ± 4.81	44.86 ± 4.96	47.95 ± 6.15	43.79 ± 5.56
(**b**) Classification results for DS2: Brain data acquired from fNIRS appended with predicted emotion scores on participant’s expression data.					
**Classifier**	**Features**	**Accuracy**	**Precision**	**Recall**	**F1-Score**
RF	ROCKET	59.93 ± 4.64	60.90 ± 7.90	59.85 ± 10.69	59.66 ± 4.80
	Statistical	51.96 ± 4.83	52.61 ± 7.91	51.49 ± 11.20	51.59 ± 4.97
XGB	ROCKET	71.55 ± 2.72	72.04 ± 5.57	72.06 ± 12.12	71.12 ± 2.74
	Statistical	51.82 ± 4.17	53.38 ± 8.15	51.10 ± 11.86	51.36 ± 4.19
SVM	ROCKET	58.69 ± 2.67	61.99 ± 11.79	58.62 ± 11.03	58.53 ± 2.83
	Statistical	46.67 ± 4.36	48.06 ± 8.94	45.57 ± 17.09	45.37 ± 4.79
kNN	ROCKET	54.63 ± 1.93	59.76 ± 16.05	54.35 ± 13.68	54.46 ± 2.06
	Statistical	41.84 ± 4.56	42.55 ± 7.61	41.54 ± 12.78	41.15 ± 4.56
GNB	ROCKET	49.21 ± 4.34	51.84 ± 10.19	48.94 ± 8.93	49.16 ± 4.29
	Statistical	34.07 ± 4.56	34.43 ± 8.16	33.81 ± 14.57	32.80 ± 4.89
FCDNN	ROCKET	91.44 ± 6.32	91.66 ± 6.24	91.44 ± 6.32	91.48 ± 6.28
	Statistical	66.00 ± 7.08	65.69 ± 7.14	66.85 ± 7.13	65.65 ± 7.20
DCAE	ROCKET	91.43 ± 9.97	91.47 ± 9.88	91.60 ± 12.73	91.48 ± 7.78
	Statistical	69.62 ± 10.80	69.25 ± 10.97	71.17 ± 11.50	69.01 ± 11.86

**Table 4:** (**a**) One-tailed Kruskal-Wallis test performed to test $H0_{1}$ for all classifer’s F1-scores compared with F1-scores of a baseline classifier [41]. (**b**) One-tailed Kruskal-Wallis test performed to test $H0_{2}$ on the distribution of F1-scores for each classifier for both DS1 and DS2.

(**a**) $H0_{1}$ tested on the distribution of F1-scores			
**Classifier**	**Dataset**	***p*** **-Value**	$H0_{1}$
RF	DS1	0.08	Accepted
XGB	DS1	p<0.05	Rejected
SVM	DS1	p<0.05	Rejected
kNN	DS1	p<0.05	Rejected
GNB	DS1	p<0.05	Rejected
FCDNN	DS1	p<0.05	Rejected
DCAE	DS1	p<0.05	Rejected
(**b**) $H0_{2}$ tested on the distribution of F1-scores			
**Classifier**	***p*** **-Value**	$H0_{2}$	
RF	*p*< 0.05	Rejected
XGB	*p*< 0.05	Rejected	
SVM	*p*< 0.05	Rejected	
kNN	*p*< 0.05	Rejected	
GNB	0.1494	Accepted	
FCDNN	0.1698	Accepted	
DCAE	0.0812	Accepted	

**Table 5:** Multiple comparison using Kruskal-Wallis test to establish $H0_{3}$ of F1-scores for all classifiers with (**a**) DS1 and (**b**) DS2.

(**a**)					
**Classifier**	**Classifier**	***p*** **-Value**	**Lower Bound**	**Estimate**	**Upper Bound**
RF	XGB	1.0000	−48.95	12.34	73.63
	SVM	*p*< 0.05	−129.55	−68.26	−6.97
	kNN	*p*< 0.05	−129.15	−67.86	−6.57
	GNB	0.6197	−20.75	40.54	101.83
	FCDNN	*p*< 0.05	−232.12	−170.83	−109.54
	DCAE	*p*< 0.05	−237.44	−176.15	−114.86
XGB	RF	1.0000	−48.95	12.34	73.63
	SVM	*p*< 0.05	−141.89	−80.60	−19.31
	kNN	*p*< 0.05	−141.45	−80.20	−18.91
	GNB	0.9763	−33.09	28.20	89.49
	FCDNN	*p*< 0.05	−244.46	−183.17	−121.88
	DCAE	*p*< 0.05	−249.78	−188.49	−127.20
SVM	RF	*p*< 0.05	−129.55	−68.26	−6.97
	XGB	*p*< 0.05	−141.89	−80.60	−19.31
	kNN	1.0000	−60.89	0.40	61.69
	GNB	*p*< 0.05	47.51	108.80	170.09
	FCDNN	*p*< 0.05	−163.86	−102.57	−41.28
	DCAE	*p*< 0.05	−169.18	−107.89	−46.60
kNN	RF	*p*< 0.05	−129.15	−67.86	−6.57
	XGB	*p*< 0.05	−141.45	−80.20	−18.91
	SVM	1.0000	−60.89	0.40	61.69
	GNB	*p*< 0.05	47.11	108.40	169.69
	FCDNN	*p*< 0.05	−164.26	−102.97	−41.68
	DCAE	*p*< 0.05	−169.58	−108.29	−47.00
GNB	RF	0.6197	−20.75	40.54	101.83
	XGB	0.9763	−33.09	28.20	89.49
	SVM	*p*< 0.05	47.51	108.80	170.09
	kNN	*p*< 0.05	47.11	108.40	169.69
	FCDNN	*p*< 0.05	−272.66	−211.37	−150.08
	DCAE	*p*< 0.05	−277.98	−216.69	−155.40
FCDNN	RF	*p*< 0.05	−232.12	−170.83	−109.54
	XGB	*p*< 0.05	−244.46	−183.17	−121.88
	SVM	*p*< 0.05	−163.86	−102.57	−41.28
	kNN	*p*< 0.05	−164.26	−102.97	−41.68
	GNB	*p*< 0.05	−272.66	−211.37	−150.08
	DCAE	1.0000	−66.61	−5.32	55.97
DCAE	RF	*p*< 0.05	−237.44	−176.15	−114.86
	XGB	*p*< 0.05	−249.78	−188.49	−127.20
	SVM	*p*< 0.05	−169.18	−107.89	−46.60
	kNN	*p*< 0.05	−169.58	−108.29	−47.00
	GNB	*p*< 0.05	−277.98	−216.69	−155.40
	FCDNN	1.0000	−66.61	−5.32	55.97
(**b**)					
**Classifier**	**Classifier**	***p*** **-Value**	**Lower Bound**	**Estimate**	**Upper Bound**
RF	XGB	*p*< 0.05	−156.77	−95.54	−34.31
	SVM	1.0000	−54.37	6.86	68.09
	kNN	*p*< 0.05	6.23	67.46	128.69
	GNB	*p*< 0.05	54.23	115.46	176.69
	FCDNN	*p*< 0.05	−199.34	−138.11	−76.88
	DCAE	*p*< 0.05	−203.14	−141.91	−80.68
XGB	RF	*p*< 0.05	−156.77	−95.54	−34.31
	SVM	*p*< 0.05	41.17	102.40	163.63
	kNN	*p*< 0.05	101.77	163.00	224.23
	GNB	*p*< 0.05	149.77	211.00	272.23
	FCDNN	0.5278	−103.80	−42.57	18.66
	DCAE	0.3694	−107.60	−46.37	14.86
SVM	RF	1.0000	−54.37	6.86	68.09
	XGB	*p*< 0.05	41.17	102.40	163.63
	kNN	0.0552	−0.63	60.60	121.83
	GNB	*p*< 0.05	47.37	108.60	169.83
	FCDNN	*p*< 0.05	−206.20	−144.97	−83.74
	DCAE	*p*< 0.05	−210.00	−148.77	−87.54
kNN	RF	*p*< 0.05	6.23	67.46	128.69
	XGB	*p*< 0.05	101.77	163.00	224.23
	SVM	0.0552	−0.63	60.60	121.83
	GNB	0.3098	−13.23	48.00	109.23
	FCDNN	*p*< 0.05	−266.80	−205.57	−144.34
	DCAE	*p*< 0.05	−270.60	−209.37	−148.14
GNB	RF	*p*< 0.05	54.23	115.46	176.69
	XGB	*p*< 0.05	149.77	211.00	272.23
	SVM	*p*< 0.05	47.37	108.60	169.83
	kNN	0.3098	−13.23	48.00	109.23
	FCDNN	*p*< 0.05	−312.99	−253.43	−193.86
	DCAE	*p*< 0.05	−318.60	−257.37	−196.14
FCDNN	RF	*p*< 0.05	−199.34	−138.11	−76.88
	XGB	0.5278	−103.80	−42.57	18.66
	SVM	*p*< 0.05	−206.20	−144.97	−83.74
	kNN	*p*< 0.05	−266.39	−206.83	−147.27
	GNB	*p*< 0.05	−312.99	−253.43	−193.86
	DCAE	1.0000	−65.03	−3.80	57.43
DCAE	RF	*p*< 0.05	−203.14	−141.91	−80.68
	XGB	0.3694	−107.60	−46.37	14.86
	SVM	*p*< 0.05	−210.00	−148.77	−87.54
	kNN	*p*< 0.05	−270.60	−209.37	−148.14
	GNB	*p*< 0.05	−318.60	−257.37	−196.14
	FCDNN	1.0000	−65.03	−3.80	57.43

## Data Availability

The data are not publicly available due to privacy or ethical restrictions.
